# Components of Empathy in Children: Factor Structure of the Empathy Quotient for Children (EQ-C)

**DOI:** 10.1007/s10803-024-06649-z

**Published:** 2025-01-09

**Authors:** Rebecca Smees, Louisa J. Rinaldi, Julia Simner

**Affiliations:** 1 School of Psychology, Pevensey Building, University of Sussex, Brighton, BN1 9QJ UK; 2 Department of Psychology, University of Edinburgh, 7 George Square, Edinburgh, EH8 9JZ UK

**Keywords:** Empathy, Well-being, Special educational needs, Sensory sensitivity, Assessment

## Abstract

Empathy is multifaceted, involving sharing and understanding the emotional and mental states of others. This study investigated the factor structure of the English-language version of the Empathy Quotient for Children (EQ-C; Auyeung et al., 2009), an empathy measure previously well-validated only as a global scale. We aimed to compare children with and without educational differences (i.e., *Special Educational Needs & Disabilities*, SEND), and explored associations between empathy and sensory sensitivities across the whole sample. Based on responses from 680 parents, we analysed data from English-speaking children aged 6–12 years, via a series of factor analyses using polychoric correlation matrices and bass-ackward analysis. Empathy domain profiling for children with SEND status (versus children without SEND status) was investigated as group differences (*t tests*). Sensory sensitivities were examined via associations (correlation) and net effects (regression). We identified an optimal four-factor solution (*emotional empathy*,* social-cognitive empathy*,* negative interactions*,* antisocial behaviours)*, and robust higher order one-, two- and three-factor models. Children with SEND status displayed empathy differences across all four empathy domains (all *p* < .001). Children with greater sensory sensitivities displayed significant differences for *social-cognitive empathy* and *negative interactions* only (both *p* < .001). We demonstrated the potential utility of the English-language EQ-C as a domain-level measure of empathy. Our paper discusses how the domains align with traditional emotional and cognitive dimensions in adults and cross-culturally. Our empathy profiles can be used alongside global empathy measures for different groups of children, with and without educational differences and sensory sensitivities.

**Supplementary Information:**

The online version contains supplementary material available at 10.1007/s10803-024-06649-z.

## Introduction

### Empathy as a Multi-Domain Trait

Empathy has been defined as the “natural capacity to share and understand other’s feelings and thoughts” (Decety & Cowell, 2017). It is widely considered to be a multidimensional phenomenon incorporating, at the very least, separate emotional and cognitive domains (Jolliffe & Farrington, 2006; Lawrence et al., 2004; Raine & Chen, 2018; Vachon & Lynam, 2016; Wang & Wang, 2015) which display distinct neurological correlates (Eres et al., 2015). *Emotional empathy* is a person’s ability to share the emotional experiences of others, for example feeling upset if a friend gets hurt. It includes aspects of emotional concern, emotional accuracy and emotion sharing (Song et al., 2019). *Cognitive empathy*, on the other hand, is the ability to attribute a mental state to another. It includes the ability to mentally “walk in another person’s shoes,” and to make predictions of how others are likely to feel or act. While some measures of empathy restrict themselves to cognitive and emotional domains alone (e.g., *Interpersonal Reactivity Index*; Davis, 1980), others have incorporated a behavioural dimension (Overgaauw et al., 2017; Reid et al., 2013). *Behavioural empathy* includes appropriate empathic behavioural responses, such as comforting or prosocial behaviours. Additionally, *somatic* (or *automatic*) *empathy*, involves bodily responses to the emotions of others, such as mimicry (Ferguson, 2016), for example smiling when other people are happy (Raine & Chen, 2018).

This paper explores the multidimensional nature of empathy through an examination of children across the general population, with and without educational differences and sensory sensitivities, using one widely-used global empathy measure. We investigated the factor structure of the English language Empathy Quotient for Children (EQ-C; Auyeung et al., 2009) in order to examine domains of empathy. Our paper is the first to explore in detail the domains of empathy in children using this measure for different groups of children with and without educational differences and sensory sensitivities.

### Correlates with Empathy

Recognizing the role of empathy in children is crucial as it influences various aspects of their lives, including social interactions, behaviour, and well-being (Jolliffe & Farrington, 2004). For example, lower empathy has been linked to bullying, higher levels of callous-unemotional traits, greater aggression (Cohen & Strayer, 1996; Lovett & Sheffield, 2007; Zych et al., 2019), poorer moral judgements (Gleichgerrcht & Young, 2013), and poorer well-being (Shanafelt et al., 2005). Capturing domains of empathy in children could also be particularly useful as there is growing evidence that certain groups (e.g., those with psychopathy, schizophrenia, sensory processing disorder, and autism spectrum conditions - henceforth autism), may have unusual presentations of empathy profiles (Blair, 2005; Cosbey et al., 2012; Derntl et al., 2012; Schwenck et al., 2012; Song et al., 2019; Tavassoli et al., 2018). For example, some autistic individuals display lower cognitive empathy on empathy measures, but intact or even heightened aspects of emotional empathy (e.g., being upset when someone else is upset) (Smith, 2009; Song et al., 2019). A comparable relationship has been found within sub-clinical autistic traits in the general population. Sindermann and colleagues (Sindermann et al., 2019) found that autistic-trait severity in adults was linked with reduced *perspective taking* (cognitive empathy), lower reported *emotional concern* (emotional empathy) but heightened personal distress (emotional empathy). Differences across domains of empathy therefore appear important for understanding individual differences, and we might therefore seek to better understand these domains in children.

### Investigating the Domain Structure of the EQ-C

In the current study we explored the factor structure of the child measure EQ-C (Auyeung et al., 2009), a parent-completed scale measuring children’s empathy. Although young children are able to provide reliable reports about many aspects of their health and well-being (Ialongo et al., 2001; Smees et al., 2019; Varni et al., 2007) they can be reluctant to report truthfully about negative behaviours (Dockerell et al., 2000). Additionally, certain children (e.g., autistic children) may be less able to report socially complex abilities such as empathy (McMahon et al., 2016), or internal emotional states (Kinnaird et al., 2019). Whilst other parent-report measures are available for tapping children’s empathy, they have certain limitations. Take, for example, the parent-report 23-item *Griffin Empathy Measure* (Dadds et al., 2008). This multidimensional model of empathy, encompassing cognitive and emotional domains, has weaknesses in terms of its psychometric properties (Dadds et al., 2008). Internal consistency for the cognitive domain is low (α = 0.62), possibly reflecting the high number of cross loading items in this scale. However, although this measure has its limitations in terms of internal consistency, it identifies cognitive and emotional empathy domains. We would therefore likely expect to find in the EQ-C. Additionally, we investigated the possibility of identifying a behavioural domain, alongside the other empathy factors expected to be identified.

Our predictions about finding domains in the EQ-C were supported by the equivalent adult scale. The forerunner to the EQ-C is the adult *Empathy Quotient* (*EQ*; Baron-Cohen et al., 2004), used widely as a single scale as well as individual domains. Lawrence and colleagues’ (2004) Principle Component Analysis (PCA) of the 28 item EQ revealed a three-factor solution. Two factors aligned with previously identified *cognitive* and *emotional* empathy domains (and were named *cognitive empathy* and *emotional reactivity)*, alongside a third factor (*social skills*). The authors concluded that both the uni-dimensional and multi-domain measures were reliable and valid scales of empathy. Other cross-cultural factorial explorations of the EQ have since validated their three-factor solution via confirmatory factor analysis, concluding that the multi-dimensional solution is perhaps preferable to a uni-dimensional scale (Allison et al., 2011; Groen et al., 2015; Muncer & Ling, 2006).

In contrast to the considerable factorial explorations of the adult scale (i.e., the EQ), the child version (i.e., the EQ-C) has received less attention. The English-language version has only been validated as a uni-dimensional scale in English (Auyeung et al., 2009), although factorial explorations of the EQ-C on Chinese and Indonesian translations have been carried out (Phallapi et al., 2018; Wang et al., 2022). Phallapi et al. (2018) conducted a PCA on 20 items (of the original 27) and extracted two factors. An inspection of the items suggests the first component consists primarily of extreme behaviours (e.g., bullying other children, pulling legs off insects) while the second factor was a mixture of the remaining items. However, there are a number of methodological reasons that may have limited the domains extracted by Phallapi and colleagues. Most notably, removing just under a third of items (*n* = 7) from the scale could have made some domains unviable. Response categories were also truncated to a 3-point scale (from the original 4-point scale), which therefore reduced the variation between items. These limited response categories (less than five categories) make a PCA approach more difficult, and a non-parametric factor analytic approach may be better suited to such data. Polychoric correlations are a non-parametric alternative to Pearson’s correlations that are often used in these situations (Asún et al., 2016), and we have taken this approach here. Wang (2022), in contrast, used a polychoric approach (on a 3-point scale) and extracted three factors, which they named *cognitive empathy*, *social skills* and *affective empathy*. However, they also conducted their analysis on a reduced item set (23 items), due to low inter-item correlations. In our own analysis we included all 27 items of the EQ-C, increasing the potential utility of any factors extracted (using a 4-point scale).

In summary, we present a novel exploration of the domains of the original EQ-C using a factorial approach. The primary goal of this analysis was to determine an optimal set of domains of the EQ-C as reported by caregivers of children with and without a SEND status. Importantly, shifting focus from a unitary empathy assessment enables an exploration of empathy profiles across multiple domains. The outcomes may be of particular importance in the assessment of empathy in children represented across different populations (see below).

### Exploring Domain-level Empathy in Special Interest Groups

Another goal of this study was to explore the structure of the empathy domains for different groups of children. First, based on the optimal domain profile identified, we investigated the empathy profiles of children who have an additional educational need, due to a learning difficulty and/or a disability -- using the UK schooling category of *Special Educational Needs and Disabilities*, SEND (Department for Education and Department of Health, 2015). The SEND system in England and Wales is designed to provide educational support to children and young people (aged 0 to 25 years) who have additional needs, as laid out in the SEND code of practice 2014 (Department of Education, 2014) and the *Children and Families Act 2014* (see Department of Health, 2015). Special needs cover four main areas: (1) communication and interaction, (2) cognition and learning; (3) social, emotional, and mental health, (4) sensory (e.g., vision impairment) and/or physical. Although the SEND population is a heterogeneous sample, they are treated as a unified group for both educational purposes (e.g., overseen by a single co-ordinator in schools) and in policy. Including a sample of SEND children allows us to test non-typically developing children in sufficient numbers (against a group of typically developing children), and our approach is validated by the meaningful group-wise differences shown in SEND groups elsewhere (Gaspar et al., 2016; Schwab, 2019).

In addition, we also investigated how differences in empathy profiles play a role in the trait of *sensory sensitivity*. Sensory sensitivity is characterised by over- (*hyper*) or under- (*hypo*) responding to sensory stimuli (Baranek et al., 2006) and can occur within a number of different sense domains (e.g., *visual*, *auditory*, *olfactory*, *gustatory*, *tactile*, *vestibular*, *proprioceptive*). *Hyper-sensitivity* typically incorporates sensory overload and avoidance behaviours, while *hypo-sensitivity* incorporates sensory under-responsivity and seeking behaviours (Dunn et al., 2016, Smees et al., 2023). For example, a person with hyper-sensitivity might find strong smells overwhelming (i.e., have sensory overload) and avoid them (i.e., exhibit sensory avoidance), while a person with hypo-sensitivity might fail to notice strong smells at all (i.e., have sensory under-responsivity or ‘dampening’) and actively seek them out (i.e., sensory seeking behaviour). Recognizing how children with sensory sensitivities experience and express empathy could provide important insights into the links between sensory processes and social interactions (Dunn et al., 2016; Bundy et al., 2007). For example, sensory sensitivities often manifest as sensory overload, and this overstimulation could impact on a child’s ability to engage in social interactions or accurately interpret emotional signals from others. Understanding more about the link between the two could enable effective interventions and support strategies (Hummerstone & Parsons., 2023). For example, classroom environments that minimize sensory triggers may improve a child’s ability to engage in social interactions and empathize with others.

We hypothesize the EQ-C will show a domain structure of more than one dimension, minimally centring around cognitive and emotional dimensions, but potentially with additional dimensions linked to social skills or behaviours. We hypothesize that children with a SEND status will exhibit different patterns of empathy compared to children who are typically developing. We also predicted empathy differences related to sensory sensitivities. Sensory sensitivities are also closely tied to autism (American Psychiatric Association, 2013; Baranek et al., 2006; Marco et al., 2011; Robertson & Simmons, 2013; Tomchek & Dunn, 2007) and correlate strongly with autistic traits in the general population (*r* = .78: Robertson & Simmons, 2013). We predict that elevated sensory sensitivities will co-occur with lower reported cognitive empathy but not lower emotional empathy (i.e., emotional empathy will be average or even elevated) as this would therefore mirror the empathy profile of autism, in which sensory sensitivities are especially common.

## Methods

### Participants

Our participants were 680 parents of children aged 6–12 years (mean age = 9.02, range 6.27–12.35, S.D. = 1.32; 47.1% female). These children had been recruited as part of the Multisense project, a longitudinal study of childhood development involving 3690 primary school children in the south-east of England (e.g., Rinaldi et al., 2020). The Multisense child cohort represented the entire student body of school years 2–5^1^ (when first tested) across 22 schools with only 1% opt-outs. Schools were state-maintained infant, junior or primary (infant + junior) schools whose mean socio-economic advantage/disadvantage was in line with the national average (measured in terms of the UK metric of Free School Meals; mean = 13.4%, range 0.7–38.1%; Taylor, 2018). Every parent of children in the cohort was invited to fill out a questionnaire about their child. We received 680 responses for the present measures (see below; which were delivered as a single measure comprising multiple questionnaires in different sections). Ethical approval was gained from the local university ethics board (the University’s Science and Technology Research Ethics Committee). Table 1 shows basic demographic details for the sample by Special Educational Needs and Disability status (SEND). SEND was classified as a binary yes/no categorisation (i.e., children were identified as having a SEND status or not, but the *type* of special need was not collected, given that resultant sub-groups would have had too small sample sizes).

From our SEND analyses (*n* = 669, mean age at time questionnaire = 9.03, range 6.27–12.35, S.D. = 1.33; 47.4% female), 45 children had a SEND status, 624 children had no SEND status and 11 children’s status was unknown (parents preferred not to say and excluded from further analyses relating to SEND). We did not collect data on autism status (i.e., whether children had any formal diagnosis of autism). Our measure of sensory sensitivities (*rGSQ-P*; Smees et al., 2021) was a continuous scale, suitable for children with and without special educational needs and disabilities across the general population. The scale is able to identify sensitivities ranging from low to high, in line with other autism-related traits (Haraguchi et al., 2019; Saito et al., 2017; Uren et al., 2019; Vital et al., 2009). There were no formal categorical cut-offs (e.g., to distinguish children with typical sensory sensitivities vs. autism). In total, 17 parents did not complete the sensory sensitivities measure, so samples were marginally smaller for our analyses involving sensory sensitivities (*n* = 663, 47.1% female; mean age at time questionnaire = 9.04, range 6.32–12.32, S.D. = 1.32).

**Table 1:** Descriptive breakdown of the sample by Special needs Status

Demographic	Control		Special needs		No Special needs information	
*Gender* *female* *male*	n	%	n	%	n	%
	302	48.3	15	33.3	3	23.7
	322	51.7	30	66.7	8	76.3
*n*	624		45		11	
*Age*	Mean	SD	Mean	SD	Mean	SD
	9.24	1.36	9.02	1.32	9.06	0.42

### Materials and Procedure

Parents completed our questionnaires either via paper copy (delivered to their school) or via our online testing portal (via a link provided to them in an email). The choice of paper versus online was dictated simply by how each school regularly communicated with its parents, and both versions were identical in all other ways. The questionnaires were presented in the order shown below, and took approximately 20 min to complete.

#### The Empathy Quotient for Children (EQ-C)

The EQ-C (Auyeung et al., 2009) is a 27 item questionnaire completed by parents about their children, and originally constructed as a single domain scale (Auyeung et al., 2009). Responses are given on a 4-point Likert scale (*definitely agree*, *slightly agree*, *slightly disagree*, *definitely disagree*), with reverse coding for *non*-empathic behaviours (e.g., “has been in trouble for physical bullying”). We scored these responses 1–4 for our main analyses, but an alternative scoring method is to truncate scores as 1–3 (where *slightly disagree* and *definitely disagree* are both scored 1). We provide both scoring methods here for completeness (see our Analytic Plan, and Supplementary Information [SI]).

The EQ-C has excellent reliability as a global measure (α = 0.93; Auyeung et al., 2009), and was originally adapted from the adult EQ (Baron-Cohen & Wheelwright, 2004) with some items newly added, but others adapted from the original (e.g., “I find it difficult to explain to others things that I understand easily, when they don’t understand it first time” was adapted for children to become “At school, when my child understands something they can easily explain it clearly to others”). In total, Auyeung el al. developed seven additional items for the purpose of tapping into the extreme ends of the empathy-spectrum, and these questions ask about relatively rare behaviours (such as bullying, or reactions to the death of a movie character).

#### The Parent-completed Glasgow Sensory Questionnaire, Reduced form (rGSQ-P)

The rGSQ-P (Smees et al., 2021) is a 24-item parent-report questionnaire assessing sensory sensitivities in children across six sense domains (i.e., visual, auditory, gustatory, olfactory, tactile, vestibular). Adapted from the adult version (GSQ; Robertson & Simmons, 2013), the rGSQ-P has 12 items measuring hyper-sensitivity (e.g., “Does your child dislike loud noises?”) and 12 items measuring hypo-sensitivity (e.g., “Does your child ever complain of having a weak sense of taste?”). There are five possible responses, on a 5-point Likert scale (0 = never, 1 = rarely, 2 = sometimes, 3 = often, 4 = always). Both the overall scale and the individual *hyper-* and *hypo-* sensitivity sub-scales show excellent reliability in children (total α = 0.87, hyper-sensitivity α = 0.85, hypo-sensitivity α = 0.77; Smees et al., 2021).

#### Special Educational Needs & Disabilities (SEND) Questionnaire

As noted above, SEND status signifies that a child has a recognised learning difficulty and/or a disability that requires additional support in school, including multiple conditions such as learning delays, autism, sensory impairment, and mental health problems (e.g., can include anxiety). Using an in-house SEND question, we elicited a fixed choice response (yes/no/rather not say) as to whether the child had a SEND status.

### Analytic Plan

We investigated the factor structure of EQ-C on the full sample (*n* = 680), applying polychoric correlations and principle axis factoring, using a rotation estimation that assumed the factors would be correlated (oblique rotation: Oblimin). Parallel analysis (PA) was used as a complimentary validation approach (Thompson & Daniel, 1996). Kaiser criterion (extracting all eigenvalues above 1) has a tendency to over-extract factors so we supplemented this with parallel analysis and internal consistency estimates to decide upon an optimal solution. As an additional exploratory investigation we employed bass-ackward methodology (Goldberg, 2006), involving a number of forced multiple factor solutions (starting at the single factor, to a two-factor solution, to three-factor etc.) to produce a hierarchical factor structure. Lastly, we explored empathy differences for children with and without SEND status (for children with SEND information available, *n* = 669) via *t*-tests and binomial regression, and the association between empathy domains and sensory sensitivities (for children with *rGSQ-P* information available, *n* = 663) via Spearman’s *Rho* correlations (due to non-normality and short ordinal scales) and linear regression model.

As noted above, the original 4-point scoring for the EQ-C was used for the factor analysis presented in the manuscript, while a factor analysis for the truncated scoring (3-point scale; see above) is shown in our SI. Analyses were carried out in SPPS 24.0 where possible. Polychoric analyses not available in SPSS were carried out using R 3.6.1, using the “polychor” package.

## Results

**Fig. 1:**
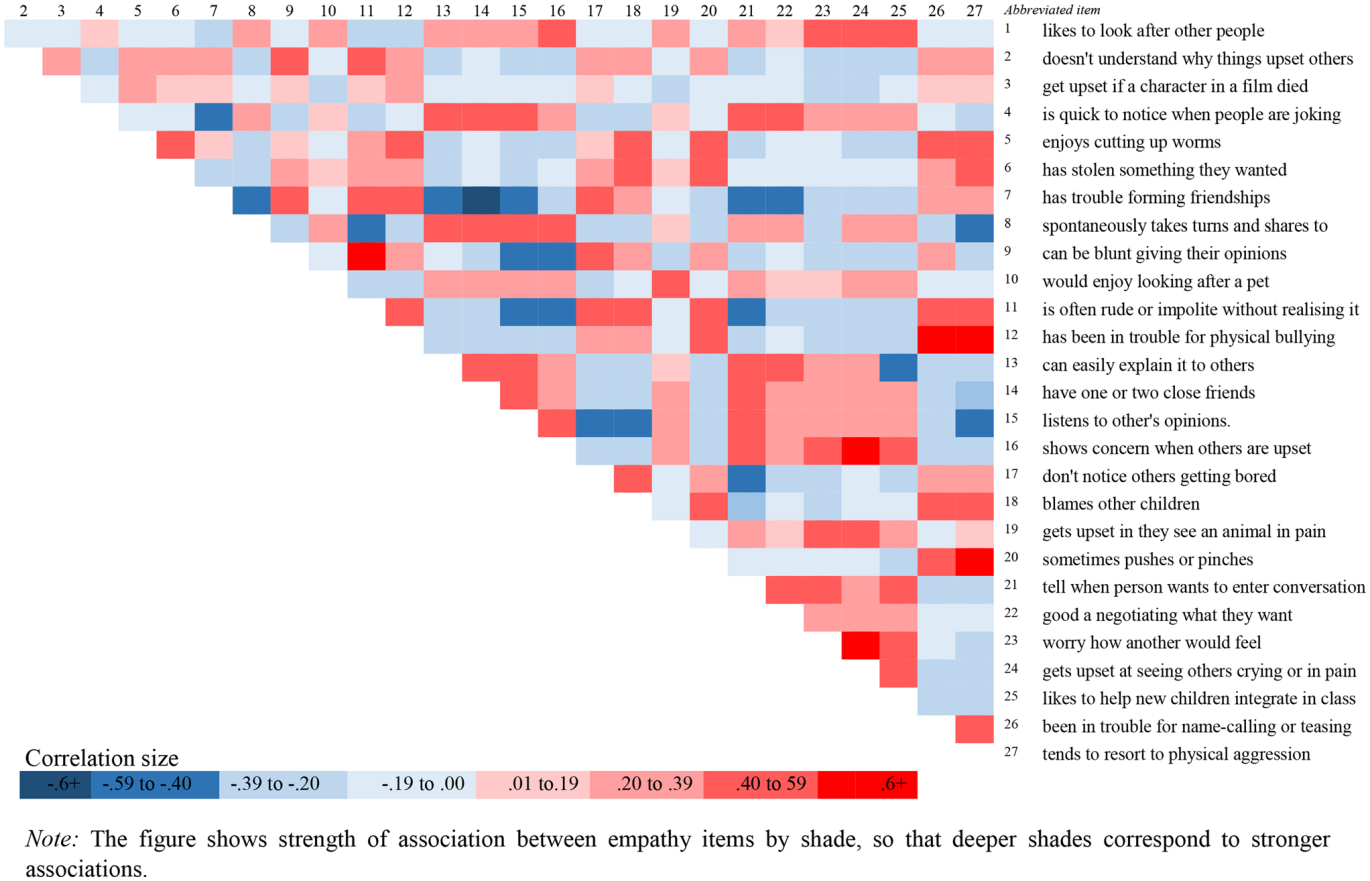
Correlation heat map for items of the EQ-C

**Fig. 2:**
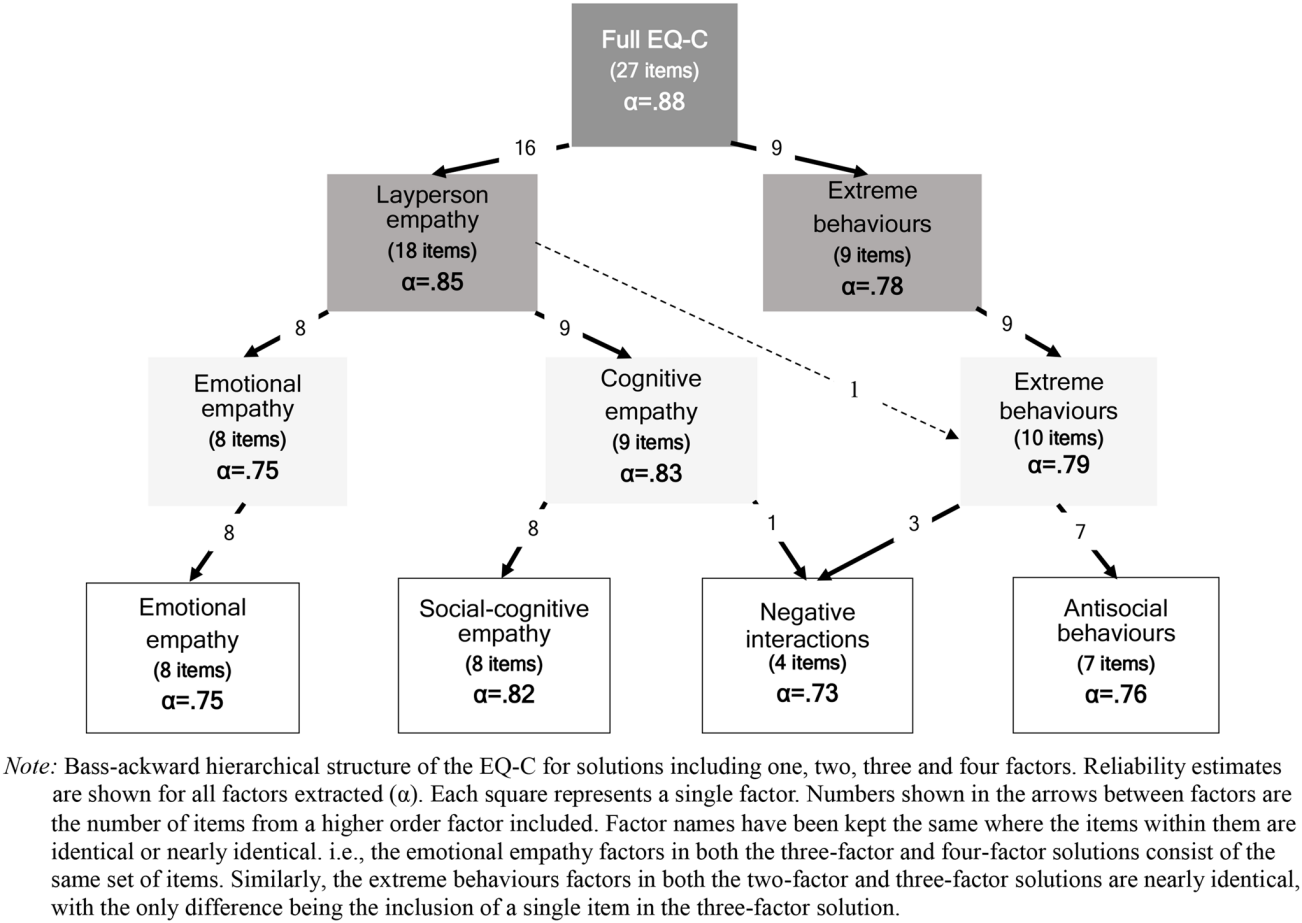
Hierarchical structure of the EQ-C using bass-ackward methodology

The aim of the paper was to explore the domain structure of the EQ-C in children age 6–12 years, also investigating how any domains identified might manifest in children with educational differences or sensory sensitivities. Although exploratory in nature, the literature suggested we were likely to identify cognitive and emotional empathy domains. In addition, we expected children with a SEND status and/or those with higher levels of sensory sensitivities would exhibit empathy profiles that were different compared to other children (i.e., those without SEND status, or those with fewer sensory sensitivities). The results section addresses the domain structure first, and Fig. 1 displays the correlations between the questions that make up the EQ-C. Table 2 presents the factor structure considered to be optimal (four-factor solution), whilst Table 3 presents the correlations between the domains identified. The final part of the domain structure investigation identified one, two, three and four domain solutions using a data driven approach (back-assward analysis, see Fig. 2). Lastly, Tables 4 and 5 detail empathy profiles for children with a SEND status and/or those with higher levels of sensory sensitivities.

### The Domain Structure of the EQ-C

#### Identifying an Optimal Factor Structure for the EQ-C

Figure 1  displays the strength of inter-item correlations between all 27 EQ-C items using a heatmap presentation. We can see from Fig. 1 that inter-item correlations are sufficiently high to make a factor analysis successful (average inter-item *r* = .30; Tabachnick & Fidell, 2014) but also that no items are too highly correlated (i.e., we can confirm that the greatest correlations, seen as the deepest colours in the heat map, do not reach the upper permissible threshold of 0.7).

The Kaiser-Meyer-Olkin (KMO) statistic test was “meritorious” (Kaiser, 1974), KMO = 0.872 (truncated KMO = 0.828), indicating the data were suitable for factor analysis (Field, 2013). Communalities, assessing the strength of the relationship of individual items with other items in the scale (higher communality = stronger relationship) ranged from 0.353 to 0.737. All items were therefore acceptable for including in the subsequent factor analysis (Child, 2006). The Kaiser criterion (eigenvalues above 1) identified six factors, of which four displayed good/excellent internal consistency (whilst the remaining two displayed poor internal consistency). Whilst the parallel analysis suggested retaining three, the four-factor solution contained the most information. This was particularly useful for investigating special populations, so we investigated this solution in the body of this paper. We have provided details of the robust three-factor solution in the SI. The three-factor solution includes *emotional empathy*, *cognitive empathy* and *extreme behaviours* (see Fig. 2) and is an alternative choice of factor structure rather than inferior. It is placed in the SI for clarity. The final forced four-factor solution identified the following factors: (a) *social-cognitive empathy* (F1 e.g., understanding the rules of social situations), (b) *antisocial behaviours* (F2, e.g., bullying or harming others), (c) *emotional empathy* (F3; e.g., feeling upset when others are upset), and (d) *negative interactions* (F4 e.g., failing to notice one’s negative impact on others). The factors produced scores where a higher score reflects greater empathy for *social-cognitive empathy* and *emotional empathy* and where lower scores reflected greater empathy for *negative interactions* and *antisocial behaviours.* See Table 2 for details.

**Table 2:** Forced four factor solution from a polychoric matrix (4-point scale)

Item My child….	F1	F2	F3	F4
has one or two close friends, as well as several other friends	0.65			
understands something they can easily explain it clearly to others [At school when]	0.65			
is good at negotiating what they want	0.64			
can easily tell when another person wants to enter into conversation with them	0.62			− 0.26
has trouble forming friendships	− 0.58			
is quick to notice when people are joking	0.58			
listens to other’s opinions even when they are different from their own	0.50	− 0.23		
when playing with other children, my child spontaneously takes turns and shares toys	0.36	− 0.30		
sometimes pushes or pinches someone if they are annoying them		0.73		
tends to resort to physical aggression to get what they want		0.72		
has been in trouble for physical bullying		0.71		
enjoys cutting up worms, or pulling the legs off insects		0.69		
has been in trouble for name-calling or teasing		0.68		
has stolen something they wanted from their sibling or friend		0.61		
blames other children for things that they themselves have done		0.60		0.23
gets upset at seeing others crying or in pain			− 0.88	
gets upset in they see an animal in pain			− 0.66	
would worry about how another would feel if they weren’t invited to a party			− 0.60	
shows concern when others are upset	0.24		− 0.53	
likes to help new children integrate in class	0.36		− 0.52	
likes to look after other people			− 0.50	
would not cry or get upset if a character in a film died	0.24		0.46	
would enjoy looking after a pet			− 0.34	
can be blunt giving their opinions, even when these may upset someone				0.78
is often rude or impolite without realising it		0.20		0.60
can seem so preoccupied with their own thoughts that they don’t notice others getting bored	− 0.24			0.51
often doesn’t understand why things upset other people so much				0.44
Cronbach alpha α	0.82	0.76	0.75	0.73

*Note*: Column 1 shows the questionnaire item. Columns 2–5 show the factor loadings for factors 1–4 (F1 social-cognitive empathy, F2 antisocial behaviours, F3 emotional empathy, F4 negative interactions) with factor loadings below 0.2 suppressed

#### Relationship between the Factors

Correlations between the four empathy factors are shown in Table 3, revealing distinct domains. The strongest correlation being between *cognitive empathy* and *negative interactions* (*r*_*s*_ = − 0.49, *p* < .001).

**Table 3:** Inter-correlations between factors in the four-factor solution

Factor	1.	2.	3.	4.
*1. Social-Cognitive empathy*	1	− 0.36	0.41	− 0.49
*2. Antisocial behaviours*		1	− 0.23	− 0.44
*3. Emotional empathy*			1	− 0.40
*4. Negative interactions*				1

*Note*. All significant at *p* < .001 level; Correlations are Spearman’s *Rho*; factors calculated from 4-point scoring system

#### Investing the Hierarchical Structure of the EQ-C

Lastly, following bass-ackward methodology (Goldberg, 2006), we compared four forced solutions (Oblimin rotation), starting from the un-rotated solution (single factor) up to the previously identified four–factor solution to explore the stability of the factors across the hierarchical structure. The resulting factor solutions are shown as a hierarchy in Fig. 2 and the full models showing factor loadings and reliability can be found in the SI. Each box in Fig. 2 represents a factor, and each level in the hierarchy represents a different factor solution. The two-factor solution split the EQ-C into a general empathy domain (we termed ‘layperson empathy’) and what we described above as ‘extreme behaviours’ (e.g., bullying). The three-factor solution is also shown in Fig. 2, which maintains the latter factor, but splits the layperson factor into two core empathy domains (emotional and cognitive empathy). Finally, Fig. 2 describes the preferred four-factor solution (i.e., *emotional empathy*, *social-cognitive empathy*, *negative interactions* and *antisocial behaviours*). We point out that the equivalent factor analyses on the 3-point scale produced largely similar results with the same hierarchical factor structure as shown in Fig. 2 emerging from the data (see SI for full details).

### Empathy Domains in Special Populations: Children with Sensory Sensitivity and SEND Status

Next, we tested how children with SEND status (special educational need or disability) scored using the domains of empathy extracted from the four-factor solution, removing those whose parents preferred not to reveal their status. Children whose parents reported no SEND status were the comparison group, against which children with SEND status were compared. Children with a SEND status presented with poorer empathy the comparison children across all four domains, i.e., lower *social-cognitive empathy t(46.4)* = -8.71, *p* < .001; lower *emotional empathy t(667)* = -7.94, *p* < .001; higher *negative interactions t(667)* = 7.65, *p* < .001; and higher *antisocial behaviours t(46.9)* = 4.78, *p* < .001. The four domains were also tested in combination within a Binomial regression model (Table 4). Results revealed *social-cognitive empathy* to be the strongest predictor of SEND status (*Exp (β)* = 0.12, *p* < .001), followed by *emotional empathy* (*Exp (β)* = 0.41, *p* = .026), explaining 41% of the variance in SEND status (using Nagelkerke’s R Square statistic; Nagelkerke, 1991). The remaining empathy domains failed to predict SEND status (*antisocial behaviours Exp (β) =* 1.65, *p* = .115; *negative interactions Exp (β)* = 1.19, *p* = .605).

**Table 4:** Binomial regression model predicting SEND status from the four-factor empathy solution

Predictor	β	SE	*p*	Exp (β)	95% CI lower	95% CI upper	Nagelkerke *R* square
Constant	-3.71	0.28	< 0.001***	0.02			0.41
*Social-cognitive emp.*	-2.10	0.37	< 0.001***	0.12	0.06	0.25	
*Antisocial behaviours*	0.50	0.32	0.115	1.65	0.88	3.09	
*Emotional empathy*	-0.90	0.40	0.026*	0.41	0.18	0.90	
*Negative interactions*	0.17	0.33	0.605	1.19	0.62	2.26	
n	669						

*Note*. All covariates Grand Mean Centred; Children with no Special Need vs. children with a Special Need (Reference group = Children with no Special Need); β Beta coefficient; SE standard Error (SE); Exp (β)) odds ratio; 95% CI confidence interval; * *p* < .05, ** *p* < .01, *** *p* < .001

We then considered the trait of sensory sensitivity, measured in our study by the *rGSQ-P* (Smees et al., 2023). We examined how sensory sensitivity aligned with each of the empathy domains identified in the four-factor solution in the current study. We remind the reader that total sensory sensitivity was calculated by summing scores from 24 items which measured hyper- and hypo- sensitivity across six sensory domains (Smees et al., 2023). To examine the relationship between empathy and sensory sensitivities, we kept empathy domains and sensory sensitivities as continuous scales (ranging from 0 to 108 for the full EQ-C 0–96 for the full *rGSP-Q*), as no official cut-off for the *rGSQ-P* exists at present. Moreover, the benefit of using continuous scales is that it reflects the relationship between two traits known to vary along a continuum across the population.

Assessing the univariate associations, we found a significant relationship between sensory sensitivities and all four empathy domains, with greater empathy associated with lower sensory sensitivity (*social-cognitive empathy r*_*s*_ = − 0.39, *p* < .001; *emotional empathy r*_*s*_ = − 0.09, *p* < .001; *negative interactions r*_*s*_ = 0.39, *p* < .001; *antisocial behaviours r*_*s*_ = 0.28, *p* < .001). However, the relationship between sensory sensitivity and *emotional empathy* was extremely small (*r*_*s*_ = − 0.09). When tested in combination (i.e., including all four domains together within the same regression model to predict sensory sensitivity), the four domains explained 22% of variance in sensory sensitivity (i.e., in total *rGSQ-P* sensory sensitivity scores; see Table 5). All four domains predicted sensitivity, although two demonstrated the strongest effects (*social-cognitive empathy β* = -0.29, *p* < .001; *negative interactions β* = 0.26, *p* < .001). *Antisocial behaviours* also predicted greater sensitivity (*β* = 0.08, *p* = .037), but should be considered of lesser importance due to small effect size. Lastly, a conditional effect emerged, with a shift in the association between *emotional empathy* and sensory sensitivity. The initial negative relationship (i.e., higher sensory sensitivity linked to lower *emotional empathy*), was reversed so that higher sensory sensitivity was linked to higher *emotional empathy* (*β* = 0.15, *p* < .001). This suggests that, when compared to children with similar empathy across the other domains, children with greater *emotional empathy* displayed greater sensory sensitivity.

**Table 5:** Multiple regression model predicting sensory sensitivities from the four-factor empathy solution

Predictor	β	SE	*p*	β	95% CI lower	95% CI upper	Adjusted *R* square
Constant	21.26	0.42	< 0.001***		20.44	22.09	0.22
*Social-cognitive emp.*	-6.54	0.98	< 0.001***	-0.29	-8.46	-4.62	
*Antisocial behaviours*	2.02	0.97	0.037	0.08	0.13	3.92	
*Emotional empathy*	4.10	1.05	< 0.001***	0.15	2.04	6.16	
*Negative interactions*	4.51	0.75	< 0.001***	0.26	3.03	5.99	
N	663						

*Note*. All covariates Grand Mean Centred; β Beta coefficient; SE standard Error (SE); *β* Standardised Beta coefficient; 95% CI confidence interval; * *p* < .05, ** *p* < .01, *** *p* < .001

## Discussion

Our paper aimed to explore the factor structure of the EQ-C using an English sample, with the expectation of identifying empathy domains among children in middle childhood (aged 6–12 years). This included our empathy profiling of children with and without educational differences (SEND status) and with varying sensory sensitivities. We conducted a series of factor-analytic approaches (traditional, parallel analysis and bass-ackward analyses). Our polychoric analyses revealed that the EQ-C can be effectively divided into several distinct, robust dimensional structures (two-, three- and four- factors), and supports a multi-dimension conceptualization of empathy in children (Blair, 2005; Eisenberg & Straye, 1987). The four-factor solution proved to be optimal when more detailed information is required, particularly when used in special populations. Lastly, we demonstrated a unique empathy profile for SEND (Special Educational Needs and Disabilities) children and those with sensory sensitivities. Profiling of this kind (e.g., using the three- or four-factor solutions) enables a more tailored and effective approach to measuring empathy in educational, research and therapeutic settings. By understanding empathy profiles of specific populations, educators and therapists can develop more personalized strategies that cater to their specific needs. Importantly, our findings were invariant across 3- and 4-point scaling. This suggests that conceptualising empathy along a 4-point scale is robust scientifically and provides the benefit of greater variation in domain scores for researchers.

### The Empathy Domains of the EQ-C

Internal consistency for all factor solutions was good, with Cronbach’s alpha of 0.70 or above for individual factors (ranging 0.73–0.88 across the two-, three- and four-factor solutions, see Fig. 2). The three- and four-factor solutions could be particularly advantageous to differentiate emotional, cognitive or behavioural aspects of empathy, as demonstrated in adults through the use of the EQ (Groen et al., 2018; Grove et al., 2014; Pepper et al., 2019). However, the four-factor solution (*social-cognitive empathy*,* emotional empathy*,* negative interactions*,* antisocial behaviours*) was deemed superior, as it offered unique and potentially valuable information that the three-factor solution did not provide. The three- and four-factor solutions identified an overlapping behavioural dimension (called *extreme behaviours* in the three-factor model and largely overlapping with *antisocial behaviours* in the four-factor model), which measures specific aspects of empathy not previously identified in other empathy questionnaires. Rather than measuring prosocial behaviours such as comforting, these two closely related factors (*extreme behaviours* in the three factor model, *antisocial behaviours* in the four factor model) represent rare negative behaviours (e.g., pulling wings off insects). These rare negative behaviours are substantively different to those included in other measures of behavioural empathy (e.g., comforting; Dadds et al., 2008; Overgaauw et al., 2017; Raine & Chen, 2018; Vachon & Lynam, 2016) and may align with aspects of empathic sadism (Breithaupt, 2018). Rather than the child displaying empathetic or prosocial behaviours, pleasure is derived from the distress caused to others. The separation of this aspect of empathy was born out in the factor analysis. For example, questions related to bullying, using physical aggression, hurting insects, name-calling, stealing, and blaming others loaded together onto a specific behavioural domain (in both three- and four-factor models).

The three- and four-factor solutions fitted the existing literature well, suggesting that emotional, cognitive, and behavioural dimensions of empathy, although correlated, can also be conceptualised as distinct domains (Blair, 2005; Brett et al., 2023; Chrysikou & Thompson, 2016; Eisenberg & Straye, 1987; Lawrence et al., 2004; Muncer & Ling, 2006). Our four-factor solution extracted an additional potential useful domain: *negative interactions*, with the remaining three factors being only minimally altered from the three-factor solution. The items within the newly identified *negative interactions* domain pertain to the processing of others’ negative reactions, such as not noticing how their bluntness may cause upset, or overlooking signs of boredom in others. This domain appears to offer distinctive insights into children’s interaction patterns. As such, our delineation of empathy into four factors offers the potential for a more comprehensive empathy profiling, suitable for both research and clinical applications. Subsequent studies could explore the empathy profiles of other groups of children, like those with autism or behavioural difficulties. For instance, research has shown that adults with callous-unemotional traits display a dissociation between emotional and cognitive empathy (emotional empathy much lower than cognitive empathy), alongside antisocial behaviours (Lethbridge et al., 2017). Similar investigations in children, linking to the empathy factors identified in the four-factor model presented in this paper could contribute to a deeper understanding of callous-unemotional traits in children. The comprehensive profiling of empathy traits in the four-factor model could prove particularly beneficial in clinical settings, especially for identifying drivers of social and communication issues in children. Specific patterns of empathy have been linked to the quality of peer relationships and prosocial behaviours elsewhere (Bianchi et al., 2020; Kim et al., 2020) as well as being linked to callous-unemotional traits (Frick et al., 2021) that could be useful to guide interventions.

The two factor solution identifies a common, general empathy factor (*layperson empathy*), closely aligned with general empathy measures found in other established questionnaires (Dadds et al., 2008; Davis., 1980; Lawrence et al., 2004), as well as a unique factor that captures intentionally harmful behaviours. This is supported by our hierarchical analysis, that revealed extreme behaviours formed a relatively distinct domain from emotional and cognitive dimensions. Whereas emotional and cognitive domains came together in the two factor, the behavioural dimension remained separate. This two factor tool could be particularly useful in educational or clinical settings for helping identify children who may need additional support in developing general empathy or managing harmful impulses.

### Comparisons to Other Studies

Our study is somewhat difficult to compare with Phallapi et al. (2018) due to methodological differences, although their findings mirrored our own two-factor solution to some extent. Similarly, when we compare our work with the one other factorial study of the EQ-C using a similar analytic approach (i.e., Wang et al., 2022; participant age 6–12, *n* = 944; translated from English) this shows notable similarities, despite their study including fewer items than our own. The polychoric factor analysis conducted by Wang et al. identified a three-factor structure they termed: *social skills*, *cognitive empathy*, and *emotional empathy*. Their *social skills* factor closely aligns with our third factor labelled we labelled *extreme behaviours* (in our three-factor model), and their *cognitive empathy* and *affective empathy* factors correspond closely to our *cognitive empathy* and e*motional empathy* factors, respectively. The overall overlap suggests that certain core elements of empathic understanding are consistent across cultures, indicating a potential universal framework of empathy. Any divergence in the factor solutions may reflect small cultural differences or be a consequence of a reduced set of questions being used in Wang et al’s study.

### Developmental Empathy in Special Populations

We investigated empathy profiles for children with a SEND status (i.e., having a Special Educational Need or Disability) and children with varying degrees of sensory sensitivity, using empathy profiling from the four-factor solution. In instances where empathy differences were observed among children with a SEND status, these were predominantly centred on lower *social-cognitive empathy.* Empathy deficits were also unevenly distributed across domains for children with sensory sensitivities. Specifically, while the association between *emotional empathy* and sensory sensitivity was only trivial (though statistically significant, *r*_*s*_ = − 0.09), more robust and significant associations were identified for *social-cognitive empathy* (*r*_*s*_ = − 0.39), *negative interactions* (*r*_*s*_ = 0.39), and *antisocial behaviours* (*r*_*s*_ = 0.28). Although still modest in size, all three robust associations indicated that lower reported empathy skills were correlated with heightened sensory sensitivities. In contrast, a conditional effect in the multiple regression analyses (including all four factors simultaneously in the model) revealed that greater emotional empathy predicted increased sensory sensitivity. Conditional effects are complex to interpret but it is possible that broader effects in the other three empathy domains were masking this relationship when analysed separately. These results reinforce an expanding body of evidence and theoretical frameworks indicating that atypical populations (e.g., autism, individuals with sensory issues) exhibit distinct empathy profiles (Shalev et al., 2023; Shalev & Uzefovsky, 2020; Smith, 2006; Sindermann et al., 2019; Song et al., 2019; Tavassoli et al., 2018). This also extends the applicability of the EQ-C beyond its existing usage within normative and autistic populations (Pan et al., 2019; Tavassoli et al., 2018).

### Limitations

The present study has provided an in-depth exploration of the structure of empathy in children, as measured by the EQ-C questionnaire. Some limitations deserve mention. Firstly, future validation using confirmatory factor approaches are required to assess overall model fit. It would be useful for the two-, three- and four- factor structures to be tested via confirmatory factor analysis, since all three factor structures have potential utility within empathy research. Secondly, we acknowledge that the EQ-C is a relatively short questionnaire, potentially limiting the number of robust domains that could be extracted. Other questionnaires have found evidence, for example, of separate emotional empathy domains (e.g., emotional concern, personal distress). We also acknowledge the heterogeneity of the SEND classification as an additional limitation. Children with distinct needs, such as variations in cognition, learning, or social, emotional, and mental health, are currently grouped together. In future research, there is an opportunity to specifically target and sample from individual populations within the SEND classification, such as those with developmental delays or autism. For example, whilst we investigated the trait of sensory sensitivity, we did not have the available data to investigate the empathy profiles of diagnosed autistic children, compared to a comparison group. We plan to carry out future studies in this area. We also acknowledge that our SEND group is small, compared to our comparison group (no SEND status). It should be noted that although parent-report questionnaires have some advantages for younger children, they do not elicit the direct experience of the child. Future research could benefit from a multiple informant approach, including children within the process. Lastly, the inclusion of standardized developmental assessments would contribute to a more detailed description of participant characteristics and prove beneficial for future analyses.

## Conclusion

We identified a series of factor structures with specific domains and domains to assist in profiling empathy of children using the EQ-C. Our hierarchical exploration allows the user to choose between lower or higher-level empathy domains. The four-factor solution could be of particular use when extended profiling empathy strengths and vulnerabilities in special populations, the two- or three-factor solutions when broader domain information is required. In the present study, we identified specific empathy profiles for children with and without reported special education needs and sensory sensitivities. This approach could be extended to specific clinical groups, for example children with autism, ADHD to determine empathy profiles for those clinical groups.

## Electronic supplementary material

Below is the link to the electronic supplementary material.

Supplementary Material 1

## Footnotes

1 School years 2–5 correspond to ages 6–10 years old in England. For example, children reach their 7th birthday during Year 2. The parent questionnaire collection continued after the initial testing of the children, so some parents completed the parent questionnaire when their children were in years 6 or 7 (age 11–12).
