# Supplementary material for: Components of Empathy in Children: Factor Structure of the Empathy Quotient for Children (EQ-C)
Source: J Autism Dev Disord. 2025 Jan 9;56(6):2148–61. doi: 10.1007/s10803-024-06649-z (PMC13222201; doi:10.1007/s10803-024-06649-z)
Supplement: Supplementary file 1 — Supplementary Material 1 [file 10803_2024_6649_MOESM1_ESM.docx]

*Supplementary Information:* **Exploring the Factorial structure of the Empathy Quotient for Children (EQ-C):**

**Components of empathy in children who are typically and non-typically developing**

Figure SI.1 Polychoric correlations heatmap (*3-point scale*)

Communalities, assessing the strength of the relationship of individual items with other items in the scale (higher communality = stronger relationship) ranged 376-.824. All items were therefore acceptable for including in the subsequent factor analysis.

|  | 2 | 3 | 4 | 5 | 6 | 7 | 8 | 9 | 10 | 11 | 12 | 13 | 14 | 15 | 16 | 17 | 18 | 19 | 20 | 21 | 22 | 23 | 24 | 25 | 26 | 27 |
| --- | --- | --- | --- | --- | --- | --- | --- | --- | --- | --- | --- | --- | --- | --- | --- | --- | --- | --- | --- | --- | --- | --- | --- | --- | --- | --- |
|  |  |  |  |  |  |  |  |  |  |  |  |  |  |  |  |  |  |  |  |  |  |  |  |  |  |  |
| 2.notunderstand-R |  |  |  |  |  |  |  |  |  |  |  |  |  |  |  |  |  |  |  |  |  |  |  |  |  |  |
| 3.film-R |  |  |  |  |  |  |  |  |  |  |  |  |  |  |  |  |  |  |  |  |  |  |  |  |  |  |
| 4.joking |  |  |  |  |  |  |  |  |  |  |  |  |  |  |  |  |  |  |  |  |  |  |  |  |  |  |
| 5.worms-R |  |  |  |  |  |  |  |  |  |  |  |  |  |  |  |  |  |  |  |  |  |  |  |  |  |  |
| 6.stolen-R |  |  |  |  |  |  |  |  |  |  |  |  |  |  |  |  |  |  |  |  |  |  |  |  |  |  |
| 7.friendtrouble-R |  |  |  |  |  |  |  |  |  |  |  |  |  |  |  |  |  |  |  |  |  |  |  |  |  |  |
| 8.takesturns |  |  |  |  |  |  |  |  |  |  |  |  |  |  |  |  |  |  |  |  |  |  |  |  |  |  |
| 9.blunt-R |  |  |  |  |  |  |  |  |  |  |  |  |  |  |  |  |  |  |  |  |  |  |  |  |  |  |
| 10.pet |  |  |  |  |  |  |  |  |  |  |  |  |  |  |  |  |  |  |  |  |  |  |  |  |  |  |
| 11.rude-R |  |  |  |  |  |  |  |  |  |  |  |  |  |  |  |  |  |  |  |  |  |  |  |  |  |  |
| 12.bullying-R |  |  |  |  |  |  |  |  |  |  |  |  |  |  |  |  |  |  |  |  |  |  |  |  |  |  |
| 13.explain |  |  |  |  |  |  |  |  |  |  |  |  |  |  |  |  |  |  |  |  |  |  |  |  |  |  |
| 14.closefriends |  |  |  |  |  |  |  |  |  |  |  |  |  |  |  |  |  |  |  |  |  |  |  |  |  |  |
| 15.opinions |  |  |  |  |  |  |  |  |  |  |  |  |  |  |  |  |  | . |  |  |  |  |  |  |  |  |
| 16.concern |  |  |  |  |  |  |  |  |  |  |  |  |  |  |  |  |  |  |  |  |  |  |  |  |  |  |
| 17.preoccupied-R |  |  |  |  |  |  |  |  |  |  |  |  |  |  |  |  |  |  |  |  |  |  |  |  |  |  |
| 18.blames-R |  |  |  |  |  |  |  |  |  |  |  |  |  |  |  |  |  |  |  |  |  |  |  |  |  |  |
| 19.animalpain |  |  |  |  |  |  |  |  |  |  |  |  |  |  |  |  |  |  |  |  |  |  |  |  |  |  |
| 20.punches-R |  |  |  |  |  |  |  |  |  |  |  |  |  |  |  |  |  |  |  |  |  |  |  |  |  |  |
| 21.tellenter |  |  |  |  |  |  |  |  |  |  |  |  |  |  |  |  |  |  |  |  |  |  |  |  |  |  |
| 22.negotiating |  |  |  |  |  |  |  |  |  |  |  |  |  |  |  |  |  |  |  |  |  |  |  |  |  |  |
| 23.party |  |  |  |  |  |  |  |  |  |  |  |  |  |  |  |  |  |  |  |  |  |  |  |  |  |  |
| 24.cryingpain |  |  |  |  |  |  |  |  |  |  |  |  |  |  |  |  |  |  |  |  |  |  |  |  |  |  |
| 25.integrate |  |  |  |  |  |  |  |  |  |  |  |  |  |  |  |  |  |  |  |  |  |  |  |  |  |  |
| 26.teasing-R |  |  |  |  |  |  |  |  |  |  |  |  |  |  |  |  |  |  |  |  |  |  |  |  |  |  |
| 27.agression-R |  |  |  |  |  |  |  |  |  |  |  |  |  |  |  |  |  |  |  |  |  |  |  |  |  |  |

|  |  |  | Correlation size | .6 + | .4 to 599 | .2 to .399 | <.2 |
| --- | --- | --- | --- | --- | --- | --- | --- |

n.b. -R signifies a negatively worded item that has been reverse coded

*Factor solutions*

*Initial factor analysis*

In the un-rotated models a total 64.6% of variance was explained by six factors (split by factor: 33.4, 10.1, 7.4, 5.2, 4.5, 3.9; truncated = 65.2% of variance explained: 33.7, 10.3, 7.4, 5.1, 4.6, 4.1). Inspection of the factors extracted found two of the six factors had poor reliability (one of the two being made up of a single item).

Figure SI.2 Scree plot for six factor solution (*4-point scale*)


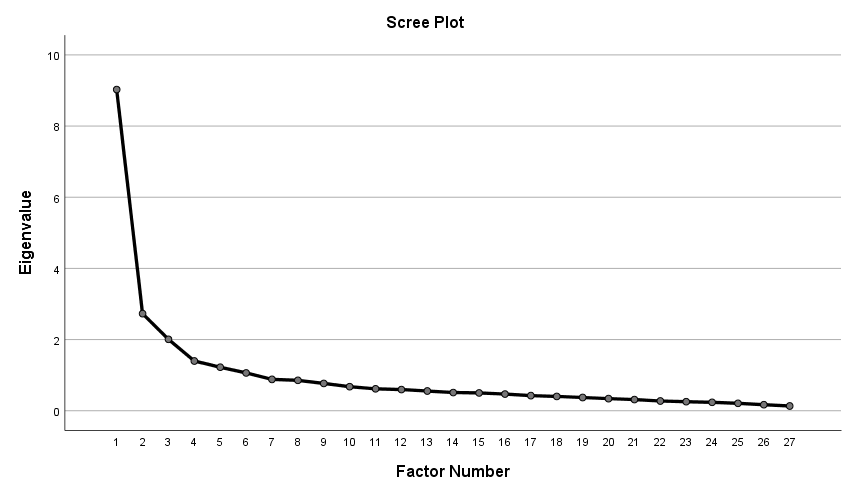


*Forced solutions*

*The alternative three-factor solution (4-point scale)*

The three-factor model extracted three robust factors as evidenced by good internal reliability (ranging .75-..83, Table SI.1), and was supported by parallel analysis. Whilst the four-factor solution provided useful information, particularly relevant to unusual populations, the three-factor solution could be considered optimal when broader empathy dimensions are required and for normative populations. Domains extracted using the forced three-factor estimation are labelled (a) *cognitive empathy* (F1 e.g., understanding the rules of social situations), (b) *extreme* *behaviours* (F2 e.g., bullying or harming others), and (c) *emotional empathy* (F3; e.g., feeling upset when others are upset). The factors overlap heavily with the four-factor solution. *Emotional empathy* is identical in the three- and four-factor solution, whilst *antisocial behaviours* (four-factor) and *extreme behaviours* (three-factor) are largely identical (*antisocial behaviours* from the four-factor solution containing one additional item).

Table. SI.1

*Three factor solution on polychoric matrix (4-point scale)*

| Item My child… | F1 | F2 | F3 |
| --- | --- | --- | --- |
| can easily tell when another person wants to enter into conversation with them | .74 |  |  |
| is quick to notice when people are joking | .67 |  |  |
| has trouble forming friendships | -.67 |  |  |
| is good a negotiating what they want | .64 |  |  |
| has one or two close friends, as well as several other friends | .64 |  |  |
| understands something they can easily explain it clearly to others [at school when] | .58 |  |  |
| listens to other's opinions even when they are different from their own | .57 | -.25 |  |
| can seem so preoccupied with their own thoughts that they don't notice others getting bored | -.44 | .20 |  |
| spontaneously takes turns and shares toys [when playing with other children] | .41 | -.31 |  |
| sometimes pushes or pinches someone if they are annoying them |  | .76 |  |
| tends to resort to physical aggression to get what they want |  | .74 |  |
| has been in trouble for name-calling or teasing |  | .72 |  |
| blames other children for things that they themselves have done |  | .69 |  |
| has been in trouble for physical bullying |  | .68 |  |
| enjoys cutting up worms, or pulling the legs off insects |  | .61 |  |
| has stolen something they wanted from their sibling or friend |  | .60 |  |
| is often rude or impolite without realising it | -.40 | .41 |  |
| can be blunt giving their opinions, even when these may upset someone | -.25 | .35 |  |
| often doesn't understand why things upset other people so much | -.20 | .24 | .21 |
| gets upset at seeing others crying or in pain |  |  | -.89 |
| gets upset in they see an animal in pain |  |  | -.67 |
| would worry about how another would feel if they weren't invited to a party | .21 |  | -.60 |
| shows concern when others are upset | .32 |  | -.54 |
| likes to help new children integrate in class | .33 |  | -.51 |
| likes to look after other people |  |  | -.50 |
| would not cry or get upset if a character in a film died | .21 |  | .47 |
| would enjoy looking after a pet |  |  | -.35 |
| α including all items | .83 | .79 | .75 |
| α excluding items loading below .40 | .81 | .76 | .76 |

Table SI.2 shows the correlations between the three factors (4-point scale), ranging in size from -.35 (*emotional empathy* and *extreme behaviours)* and -.54 (*extreme behaviours* and *social-cognitive empathy).* A higher score for *emotional empathy* and *social-cognitive empathy* represents greater empathy, whilst a higher score for *extreme behaviours* represents more extreme behaviours.

SI.2

*Inter-correlations between factors in the three-factor solution*

| Factor | 1. | 2. | 3. |
| --- | --- | --- | --- |
| *1. Emotional empathy* | 1 | -.35 | .46 |
| *2. Extreme behaviours* |  | 1 | -.54 |
| *3. Social-Cognitive empathy* |  |  | 1 |

*Note.* All significant at p < .001 level; Correlations are Spearman’s *Rho*; factors calculated from 4-point scoring system

The single factor solution, using the 4-point scale, is shown in Table. SI.3. One item, ‘would not cry or get upset if a character in a film died’ loaded weakly on this single factor.

Table. SI.3

*Single factor solution on polychoric matrix (4-point scale)*

|  | Item | F1 |
| --- | --- | --- |
| 15 | listens to other's opinions even when they are different from their own | .72 |
| 16 | shows concern when others are upset | .71 |
| 11 | is often rude or impolite without realising it | -.70 |
| 7 | has trouble forming friendships | -.66 |
| 8 | spontaneously takes turns and shares toys [when playing with other children] | .65 |
| 21 | can easily tell when another person wants to enter into conversation with them | .65 |
| 27 | tends to resort to physical aggression to get what they want | -.63 |
| 25 | likes to help new children integrate in class | .59 |
| 14 | has one or two close friends, as well as several other friends | .59 |
| 13 | understands something they can easily explain it clearly to others [at school when] | .59 |
| 12 | has been in trouble for physical bullying | -.59 |
| 18 | blames other children for things that they themselves have done | -.58 |
| 9 | can be blunt giving their opinions, even when these may upset someone | -.57 |
| 23 | would worry about how another would feel if they weren't invited to a party | .56 |
| 24 | gets upset at seeing others crying or in pain | .56 |
| 17 | can seem so preoccupied with their own thoughts that they don't notice others getting bored | -.55 |
| 20 | sometimes pushes or pinches someone if they are annoying them | -.55 |
| 26 | has been in trouble for name-calling or teasing | -.54 |
| 4 | is quick to notice when people are joking | .53 |
| 2 | often doesn't understand why things upset other people so much | -.50 |
| 22 | is good a negotiating what they want | .46 |
| 5 | enjoys cutting up worms, or pulling the legs off insects | -.45 |
| 1 | likes to look after other people | .44 |
| 6 | has stolen something they wanted from their sibling or friend | -.40 |
| 10 | would enjoy looking after a pet | .37 |
| 19 | gets upset in they see an animal in pain | .36 |
| 3 | would not cry or get upset if a character in a film died | -.22 |
|  | α including all items | .88 |
|  | α excluding items loading below .40 | .88 |

Similarly, the same item loaded weakly on F2 in the two-factor model (Table. SI.4), although improved the internal consistency of the factor.

Table. SI.4

*Two factor solution on polychoric matrix (4-point scale)*

| Item | My child…. | F1 | F2 |
| --- | --- | --- | --- |
| 24 | gets upset at seeing others crying or in pain | .77 |  |
| 16 | shows concern when others are upset | .72 |  |
| 25 | likes to help new children integrate in class | .71 |  |
| 23 | would worry about how another would feel if they weren't invited to a party | .69 |  |
| 21 | can easily tell when another person wants to enter into conversation with them | .65 |  |
| 19 | gets upset in they see an animal in pain | .57 |  |
| 1 | likes to look after other people | .57 |  |
| 22 | is good a negotiating what they want | .54 |  |
| 14 | has one or two close friends, as well as several other friends | .49 |  |
| 4 | is quick to notice when people are joking | .47 |  |
| 15 | listens to other's opinions even when they are different from their own | .47 | -.37 |
| 13 | understands something they can easily explain it clearly to others [at school when] | .47 | -.21 |
| 7 | has trouble forming friendships | -.43 | .33 |
| 10 | would enjoy looking after a pet | .39 |  |
| 8 | spontaneously takes turns and shares toys [when playing with other children] | 38 | -.38 |
| 17 | can seem so preoccupied with their own thoughts that they don't notice others getting bored ** | -.35 | .30 |
| 2 | often doesn't understand why things upset other people so much | -.33 | .26 |
| 3 | would not cry or get upset if a character in a film died | -.26 |  |
| 20 | sometimes pushes or pinches someone if they are annoying them |  | .77 |
| 27 | tends to resort to physical aggression to get what they want |  | .76 |
| 18 | blames other children for things that they themselves have done |  | .75 |
| 26 | has been in trouble for name-calling or teasing |  | .71 |
| 12 | has been in trouble for physical bullying |  | .65 |
| 6 | has stolen something they wanted from their sibling or friend |  | .62 |
| 5 | enjoys cutting up worms, or pulling the legs off insects |  | .55 |
| 11 | is often rude or impolite without realising it | -.33 | .49 |
| 9 | can be blunt giving their opinions, even when these may upset someone | -.28 | .39 |
|  | α including all items | .85 | .78 |
|  | α excluding items loading below .40 | .82 | .76 |

*The alternative 3-point scale analysis*

The factor analysis was run identically on the truncated scale (3-point).

Figure SI.3 Scree plot for six factor solution (*3- point scale*)


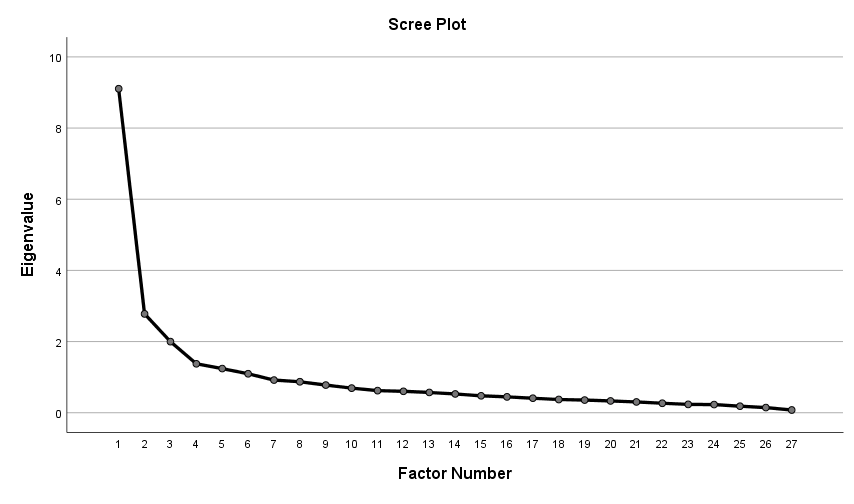


Tables SI.5-SI.8 display the forced solutions using the 3-point scale.

Table. SI.5

*Single factor solution on polychoric matrix (3-point scale)*

|  | Item | F1 |
| --- | --- | --- |
| 15 | listens to other's opinions even when they are different from their own | .72 |
| 16 | shows concern when others are upset | .71 |
| 11 | is often rude or impolite without realising it-R | .69 |
| 8 | spontaneously takes turns and shares toys [when playing with other children] | .68 |
| 7 | has trouble forming friendships -R | .66 |
| 21 | can easily tell when another person wants to enter into conversation with them | .65 |
| 27 | tends to resort to physical aggression to get what they want-R | .62 |
| 14 | has one or two close friends, as well as several other friends | .61 |
| 25 | likes to help new children integrate in class | .59 |
| 17 | can seem so preoccupied with their own thoughts that they don't notice others getting bored -R | .59 |
| 13 | understands something they can easily explain it clearly to others [at school when] | .58 |
| 12 | has been in trouble for physical bullying-R | .58 |
| 23 | would worry about how another would feel if they weren't invited to a party | .58 |
| 18 | blames other children for things that they themselves have done-R | .58 |
| 9 | can be blunt giving their opinions, even when these may upset someone-R | .57 |
| 24 | gets upset at seeing others crying or in pain | .56 |
| 26 | has been in trouble for name-calling or teasing-R | .54 |
| 20 | sometimes pushes or pinches someone if they are annoying them-R | .54 |
| 2 | often doesn't understand why things upset other people so much -R | .53 |
| 4 | is quick to notice when people are joking | .53 |
| 1 | likes to look after other people | .45 |
| 22 | is good a negotiating what they want | .45 |
| 5 | enjoys cutting up worms, or pulling the legs off insects -R | .41 |
| 6 | has stolen something they wanted from their sibling or friend -R | .39 |
| 10 | would enjoy looking after a pet | .38 |
| 19 | gets upset in they see an animal in pain | .38 |
| 3 | would not cry or get upset if a character in a film died -R | .22 |

-R negatively worded items in 1-3 point scale are positively scored

Table. SI.6

*Two factor solution on polychoric matrix (3- point scale)*

| Item | My child…. | F1 | F2 |
| --- | --- | --- | --- |
| 24 | gets upset at seeing others crying or in pain | .77 |  |
| 16 | shows concern when others are upset | .74 |  |
| 23 | would worry about how another would feel if they weren't invited to a party | .70 |  |
| 25 | likes to help new children integrate in class | .69 |  |
| 21 | can easily tell when another person wants to enter into conversation with them | .64 |  |
| 19 | gets upset in they see an animal in pain | .58 |  |
| 1 | likes to look after other people | .57 |  |
| 22 | is good a negotiating what they want | .54 |  |
| 14 | has one or two close friends, as well as several other friends | .52 |  |
| 4 | is quick to notice when people are joking | .49 |  |
| 7 | has trouble forming friendships-R | .48 | -.28 |
| 15 | listens to other's opinions even when they are different from their own | .47 | -.38 |
| 13 | understands something they can easily explain it clearly to others [at school when] | .44 | -.24 |
| 10 | would enjoy looking after a pet | .41 |  |
| 2 | often doesn't understand why things upset other people so much-R | .40 | -.22 |
| 17 | can seem so preoccupied with their own thoughts that they don't notice others getting bored-R | .37 | -.31 |
| 3 | would not cry or get upset if a character in a film died-R | .27 |  |
| 20 | sometimes pushes or pinches someone if they are annoying them-R |  | -.77 |
| 27 | tends to resort to physical aggression to get what they want-R |  | -.76 |
| 26 | has been in trouble for name-calling or teasing-R |  | -.72 |
| 18 | blames other children for things that they themselves have done-R |  | -.72 |
| 12 | has been in trouble for physical bullying-R |  | -.66 |
| 6 | has stolen something they wanted from their sibling or friend-R |  | -.61 |
| 5 | enjoys cutting up worms, or pulling the legs off insects-R |  | -.53 |
| 11 | is often rude or impolite without realising it-R | .34 | -.49 |
| 8 | spontaneously takes turns and shares toys [when playing with other children] | .40 | -.40 |
| 9 | can be blunt giving their opinions, even when these may upset someone-R | .28 | -.40 |

-R negatively worded items in 1-3 point scale are positively scored

Table. SI.7

*Three factor solution on polychoric matrix (3-point scale)*

| Item | My child…. | F1 | F2 | F3 |
| --- | --- | --- | --- | --- |
| 21 | can easily tell when another person wants to enter into conversation with them | .70 |  |  |
| 14 | has one or two close friends, as well as several other friends | .70 |  |  |
| 7 | has trouble forming friendships-R | .70 |  |  |
| 4 | is quick to notice when people are joking | .67 |  |  |
| 22 | is good a negotiating what they want | .65 |  |  |
| 13 | understands something they can easily explain it clearly to others [at school when] | .57 |  |  |
| 15 | listens to other's opinions even when they are different from their own | .56 | -.28 |  |
| 17 | can seem so preoccupied with their own thoughts that they don't notice others getting bored-R | .43 | -.24 |  |
| 8 | spontaneously takes turns and shares toys [when playing with other children] | .40 | -.34 |  |
| 20 | sometimes pushes or pinches someone if they are annoying them-R |  | -.78 |  |
| 27 | tends to resort to physical aggression to get what they want-R |  | -.75 |  |
| 26 | has been in trouble for name-calling or teasing-R |  | -.74 |  |
| 12 | has been in trouble for physical bullying-R |  | -.68 |  |
| 18 | blames other children for things that they themselves have done-R |  | -.68 |  |
| 6 | has stolen something they wanted from their sibling or friend-R |  | -.60 |  |
| 5 | enjoys cutting up worms, or pulling the legs off insects-R |  | -.58 |  |
| 11 | is often rude or impolite without realising it-R | .35 | -.43 |  |
| 9 | can be blunt giving their opinions, even when these may upset someone-R |  | -.39 |  |
| 24 | gets upset at seeing others crying or in pain |  |  | .90 |
| 19 | gets upset in they see an animal in pain |  |  | .67 |
| 23 | would worry about how another would feel if they weren't invited to a party |  |  | .62 |
| 16 | shows concern when others are upset | .27 |  | .59 |
| 1 | likes to look after other people |  |  | .53 |
| 25 | likes to help new children integrate in class | .28 |  | .52 |
| 3 | would not cry or get upset if a character in a film died-R | -.21 |  | .48 |
| 10 | would enjoy looking after a pet |  |  | .35 |
| 2 | often doesn't understand why things upset other people so much-R |  | -.23 | .29 |

-R negatively worded items in 1-3 point scale are positively scored

Table. SI.8

*Four factor solution from a polychoric matrix (3- point scale)*

| Item | My child…. | F1 | F2 | F3 | F4 |
| --- | --- | --- | --- | --- | --- |
| 14 | has one or two close friends, as well as several other friends | .68 |  |  |  |
| 22 | is good at negotiating what they want | .65 |  |  |  |
| 21 | can easily tell when another person wants to enter into conversation with them | .64 |  |  |  |
| 7 | has trouble forming friendships-R | .63 |  |  |  |
| 4 | is quick to notice when people are joking | .62 |  |  |  |
| 13 | understands something they can easily explain it clearly to others [at school when] | .61 | -.21 |  |  |
| 15 | listens to other's opinions even when they are different from their own | .52 | -.25 |  |  |
| 8 | when playing with other children, my child spontaneously takes turns and shares toys | .37 | -.32 |  |  |
| 20 | sometimes pushes or pinches someone if they are annoying them-R |  | -.77 |  |  |
| 27 | tends to resort to physical aggression to get what they want-R |  | -.74 |  |  |
| 26 | has been in trouble for name-calling or teasing-R |  | -.69 |  |  |
| 12 | has been in trouble for physical bullying-R |  | -.69 |  |  |
| 5 | enjoys cutting up worms, or pulling the legs off insects-R |  | -.63 |  |  |
| 18 | blames other children for things that they themselves have done-R |  | -.61 |  |  |
| 6 | has stolen something they wanted from their sibling or friend-R |  | -.60 |  |  |
| 24 | gets upset at seeing others crying or in pain |  |  | .89 |  |
| 19 | gets upset in they see an animal in pain |  |  | .64 |  |
| 23 | would worry about how another would feel if they weren't invited to a party |  |  | .62 |  |
| 16 | shows concern when others are upset | .23 |  | .56 |  |
| 25 | likes to help new children integrate in class | .33 |  | .55 |  |
| 1 | likes to look after other people |  |  | .54 |  |
| 3 | would not cry or get upset if a character in a film died | -.23 |  | .45 |  |
| 10 | would enjoy looking after a pet |  |  | .33 |  |
| 9 | can be blunt giving their opinions, even when these may upset someone-R |  |  |  | -.74 |
| 11 | is often rude or impolite without realising it-R | .21 | -.22 |  | -.57 |
| 17 | can seem so preoccupied with their own thoughts that they don't notice others getting bored-R | .31 |  |  | -.47 |
| 2 | often doesn't understand why things upset other people so much-R |  |  | .23 | -.46 |

-R negatively worded items in 1-3 point scale are positively scored
